# Trading Places—Switching Frataxin Function by a Single Amino Acid Substitution within the [Fe-S] Cluster Assembly Scaffold

**DOI:** 10.1371/journal.pgen.1005192

**Published:** 2015-05-21

**Authors:** Dennis R. Dean, Patricia C. Dos Santos

**Affiliations:** 1 Department of Biochemistry, Virginia Tech, Blacksburg, Virginia, United States of America; 2 Department of Chemistry, Wake Forest University, Winston-Salem, North Carolina, United States of America; Universidad de Sevilla, Spain

Simple inorganic structures comprised of iron and sulfur are called [Fe-S] clusters. They likely represent one of the earliest prosthetic groups associated with the emergence of life on earth and continue to have essential roles in sustaining many metabolic processes in almost all existing life forms. For example, proteins that contain one or more [Fe-S] clusters, generally referred to as [Fe-S] proteins, are involved in a wide variety of important cellular functions, including energy transformations, catalysis, and regulation of gene expression. In recent years, the assembly of [Fe-S] clusters and their trafficking within biological systems has captured the attention of researchers because defects in the process can lead to disruption of important metabolic processes, which, in humans, is often manifested in a variety of pathological conditions [1].

**Fig 1:**
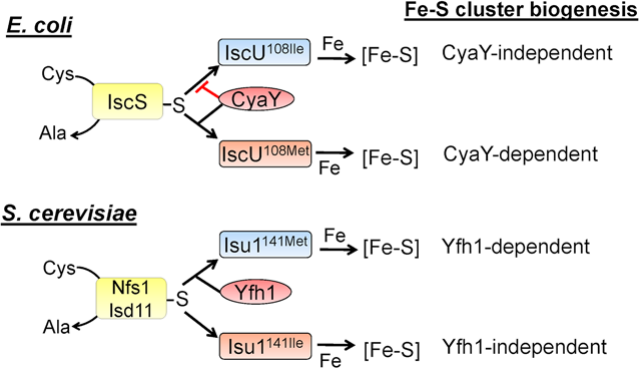
Frataxin involvement in [Fe-S] cluster biogenesis in *E*. *coli* and *S*. *cerevisiae*. Cysteine desulfurases IscS and Nfd1/Isd11 are shown in yellow, and the frataxin orthologs CyaY and Yfh1 are shown in red. The wild-type scaffold proteins IscU Ile^108^ and Isu1 Met^141^ are indicated in blue, while the variant proteins are in orange (IscU Met^108^ and Isu1 Ile^141^). In *E*. *coli*, CyaY has been shown to inhibit *in vitro* assembly of Fe-S cluster on wild-type IscU (indicated by red bar), and is required for *in vivo* [Fe-S] cluster biogenesis in strains containing IscU Met^108^ (indicated by black bar). In *S*. *cerevisiae*, Yfh1 facilitates [Fe-S] cluster assembly on the wild-type Isu1 (indicated by black bar), while a strain containing Isu1 Ile^141^ does not require Yfh1 for *in vivo* [Fe-S] cluster biogenesis.

Two central players involved in biological [Fe-S] cluster formation include an L-cysteine desulfurase (designated IscS in bacteria or Nfs1 in eukaryotes) and an assembly scaffold (designated IscU in bacteria or Isu in eukaryotes). IscS/Nfs1 delivers S in the form of an enzyme-bound persulfide to IscU/Isu upon which nascent [Fe-S] clusters are formed prior to their delivery to target proteins or intermediate carriers (Fig 1) [2–4]. Given the early evolutionary emergence of [Fe-S] clusters, as well as their critical metabolic function, it is not surprising that the primary structures and mechanistic features of the IscS/Nfs and IscU/Isu orthologs are conserved throughout nature. Nevertheless, some fundamental differences between the prokaryotic and eukaryotic systems have become apparent. For example, the eukaryotic Nfs L-cysteine desulfurase requires an additional subunit, Isd11 [5], for basal activity, but the bacterial ortholog IscS does not [6].

Another difference in prokaryotic versus eukaryotic [Fe-S] cluster assembly that has confounded the research community involves the role of a protein called Frataxin (designated Fxn in humans, Yfh1 in yeast, and CyaY in bacteria) [7,8]. Frataxin has been the subject of intense investigation for many years because defects in its formation are associated with a debilitating human neurodegenerative disease known as Friedreich’s ataxia [1]. Yeast loss of Yfh1 function is linked to dysregulation of Fe homeostasis and defects in [Fe-S] cluster formation [9]. In contrast to the important function of Yfh1 in yeast, complete loss of the bacterial ortholog, CyaY, does not exhibit a profound phenotype [10,11]. These apparently contradictory results were reconciled by biochemical analyses obtained using *in vitro* IscS-IscU or Nfs-Isd11-Isu directed [Fe-S] cluster assembly. In these studies, it was shown that the bacterial Frataxin ortholog CyaY inhibits [Fe-S] cluster assembly by slowing IscS mediated S delivery to IscU, whereas the eukaryotic Frataxin ortholog stimulates [Fe-S] cluster assembly by acceleration of Nfs/Isd11 mediated S delivery to Isu (Fig 1) [7,12,13]. Work described in the articles by Yoon et al. [14] and Roche *et al*. [15] now provide remarkable *in vivo* complements to the pioneering biochemical studies.

Although Yfh1 inactivation causes severe metabolic defects in yeast, the Dancis group was able to isolate a fast-growing strain that bypasses the Yfh1 requirement [16]. The spontaneous suppressor mutation leading to Yfh1 independence is localized within *isu1* and results in substitution of the Isu1 Met^141^ residue by Isu1 Ile^141^. This result was particularly intriguing because the *Escherichia coli* IscU residue corresponding to Isu Met^141^ is naturally occupied by IscU Ile^108^. Yoon *et al*. now report on an exhaustive study on suppression of the yeast Yfh1 deletion phenotype by using both directed mutagenesis and genetic selection strategies [14]. They find that suppression of the Yfh1 deletion phenotype can only be accomplished by substitutions at the Met^141^ position and only by substitution of an Ile, Leu, Cys, or Val residue. Remarkably, bioinformatic analyses reveal that these four amino acids are also, by far, the most highly represented and naturally occurring residues at this position among almost all IscU-containing prokaryotes. In contrast, examination of a vast number of eukaryotic Isu primary structures reveals that Isu Met^141^ is strictly conserved. A second approach that provided further evidence on the correlation between Yfh1 dependence and the Isu Met^141^ residue involved heterologous expression of the *E*. *coli* IscU in a yeast Yfh1 depletion strain. These experiments demonstrated that the wild-type *E*. *coli* IscU could be used to rescue the yeast phenotype associated with the lack of Yfh1, but the IscU Met^108^-substituted form could not.

Building on the observation that the yeast Yfh1 deletion phenotype is suppressed by substitution of Isu Met^141^ with the corresponding bacterial IscU Ile^108^ residue, Roche et al. asked the reciprocal question [15]. Namely, does substitution of the *E*. *coli* IscU Ile^108^ residue by Met^108^ render [Fe-S] cluster assembly *dependent* on the presence of CyaY, the bacterial ortholog of yeast Yfh1? The answer to that question is yes. Roche *et al*. also explored the phylogenic conservation at the IscU residue^108^ position as a way to gain clues into the evolutionary emergence of the Yfh1-dependent eukaryotic Isu form. Interestingly, it was found that the *Rickettsia* are one of very few bacteria that naturally carry an Iscu Met^108^. Given the endosymbiotic lifestyle of the *Rickettsia*, and its possible role in primordial acquisition of mitochondria, Roche et al. make the credible suggestion that the dependence of “Frataxin” for [Fe-S] cluster synthesis was acquired from bacteria, rather than independently within the established Eukaryotic lineage.

These two complementary studies have led to the amazing observation that substitution of a single amino acid carried within the ancient IscU family of proteins can lead to a profound alteration in the function of an associated accessory component, as either an activator or as an apparent inhibitor of the cluster assembly process. They also provide strong *in vivo* evidence to support a growing body of elegant biochemical studies that have demonstrated an important role for the Frataxin family of proteins in modulating intermolecular S-transfer during [Fe-S] cluster biosynthesis. Finally, they highlight the central importance of the IscU-type of molecular scaffold that, save for the effects of one amino acid variation, appears to have a structure and mechanism that has been conserved through time and throughout nature.
